# Controlled Liposome Delivery from Chitosan-Based Thermosensitive Hydrogel for Regenerative Medicine

**DOI:** 10.3390/ijms23020894

**Published:** 2022-01-14

**Authors:** Franco Furlani, Arianna Rossi, Maria Aurora Grimaudo, Giada Bassi, Elena Giusto, Filippo Molinari, Florigio Lista, Monica Montesi, Silvia Panseri

**Affiliations:** 1 National Research Council of Italy-Institute of Science and Technology for Ceramics (ISTEC-CNR), Via Granarolo 64, I-48018 Faenza, Italy; arianna.rossi@istec.cnr.it (A.R.); aurora_grimaudo@hotmail.it (M.A.G.); giada.bassi@istec.cnr.it (G.B.); elena.giusto@istec.cnr.it (E.G.); monica.montesi@istec.cnr.it (M.M.); 2 Army Medical Center, Scientific Department, I-00184 Rome, Italy; filippo.molinari@esercito.difesa.it (F.M.); florigio.lista@esercito.difesa.it (F.L.)

**Keywords:** thermosensitive hydrogel, regenerative medicine, liposome, controlled delivery, extracellular vesicles

## Abstract

This work describes the development of an injectable nanocomposite system based on a chitosan thermosensitive hydrogel combined with liposomes for regenerative medicine applications. Liposomes with good physicochemical properties are prepared and embedded within the chitosan network. The resulting nanocomposite hydrogel is able to provide a controlled release of the content from liposomes, which are able to interact with cells and be internalized. The cellular uptake is enhanced by the presence of a chitosan coating, and cells incubated with liposomes embedded within thermosensitive hydrogels displayed a higher cell uptake compared to cells incubated with liposomes alone. Furthermore, the gelation temperature of the system resulted to be equal to 32.6 °C; thus, the system can be easily injected in the target site to form a hydrogel at physiological temperature. Given the peculiar performance of the selected systems, the resulting thermosensitive hydrogels are a versatile platform and display potential applications as controlled delivery systems of liposomes for tissue regeneration.

## 1. Introduction

**Figure 1:**
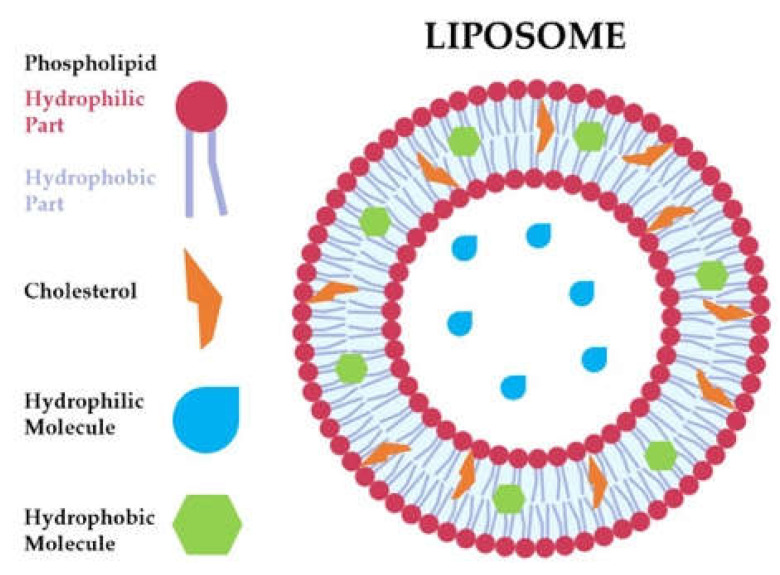
Schematic representation of liposome structure.

Liposomes are nanocarriers made of single units that can self-assemble, driven by soft interactions. Like cell membranes, they can be composed mainly of phospholipids and cholesterol (Figure 1). Phospholipids are amphiphilic macromolecules that exhibit both hydrophobic and hydrophilic behavior; thus, once in contact with water, they tend to aggregate, forming lipid bilayer nanocarriers [1]. Liposomes have some interesting advantages: they are easy to prepare, versatile, highly biocompatible, and biodegradable [2,3]. Moreover, they can be loaded with biologically active compounds that can be conveyed to a desired target. The cell–liposome interaction depends on liposomes’ physiochemical features such as size, shape, hydrophobicity, and surface charge. Specifically, neutral and negatively charged particles display poor cell interaction, whereas positively charged liposomes usually show enhanced cell interaction [3,4].

Due to liposomes’ structure, both hydrophilic and hydrophobic molecules can be loaded, entailing their use in a wide range of applications (Figure 1). First of all, they are employed as bioactive agent delivery systems [5]—for example, drugs such as doxorubicin for anticancer therapy [6] and levodopa for Parkinson’s disease treatment [7]. Moreover, the nutraceutical is an emerging field, and liposomes loaded with compounds such as ascorbic acid [8], curcumin [9], and β-carotene [10] are being widely studied [11]. Nucleic acids can also be entrapped within liposomes for gene therapy and vaccine delivery [12].

Liposomes resemble extracellular vesicles (EVs), the lipid bilayer-delimited particles naturally produced and secreted by cells. Hence, liposomes can be used as a simplified EV study model. EVs influence almost every process in cell physiology, playing a key role in intercellular communication and immunomodulation [13]. Recently, EVs emerged as promising pleiotropic tools also in regenerative medicine [14] as they offer significant benefits compared to cell therapy; since they cannot undergo neoplastic transformation, they are stable under freezing/thawing cycles, and they can be loaded with drugs and/or biomolecules [13]. EVs have recently been demonstrated in preclinical neurotrauma models, including spinal cord injury and traumatic brain injury, showing their role in functional recovery and neurovascular plasticity [15]. Additionally, EVs demonstrated an immunomodulatory function and a drug delivery function [13,16]. Unfortunately, several in vivo studies indicate that the therapeutic efficacy of both injected liposomes and EVs is largely reduced by their rapid clearance and short half-life that limit their use as an effective therapy [14,17,18]. To overcome these drawbacks, biomaterial-based strategies represent promising approaches to enhance their therapeutic potency in tissue regeneration. Indeed, biomaterials are able to control the spatial and temporal delivery of active agents with a controlled release profile, thus reducing adverse effects, improving potency, and decreasing the number and amount of doses required [19].

Recently, great efforts have been devoted to developing suitable hydrogels as controlled release systems [20,21]. Hydrogels are networks able to mimic natural the extracellular matrix (ECM), support cell adhesion, and allow the diffusion of nutrients, gases, and waste products [19]. Naturally occurring biopolymers are widely exploited to fabricate ECM-mimicking materials since they display most of the abovementioned properties. In this scenario, chitosan represents a very attractive biopolymer for drug delivery and regenerative medicine applications [20,22,23,24,25,26,27,28]. Indeed, chitosan is a low-toxicity, non-immunogenic, and biodegradable polymer deriving from the partial deacetylation of chitin, the second most abundant polysaccharide on Earth [29,30].

Herein we report an original method to form an injectable thermosensitive nanocomposite hydrogel based on chitosan for the controlled delivery of liposomes. The biocompatibility towards cell models and the performance of this set of hybrid materials are additionally disclosed, confirming the interesting role that these hydrogels could play in regenerative medicine.

## 2. Results

### 2.1. Physicochemical Characterization of Hydrogel and Liposomes

In order to promote chitosan gelation at physiological temperature, β-glycerophosphate was exploited as a crosslinking agent. β-glycerophosphate was mixed with a chitosan solution, forming a colorless liquid mixture. Upon increasing the temperature, β-glycerophosphate promoted the formation of a turbid hydrogel. 

**Figure 2:**
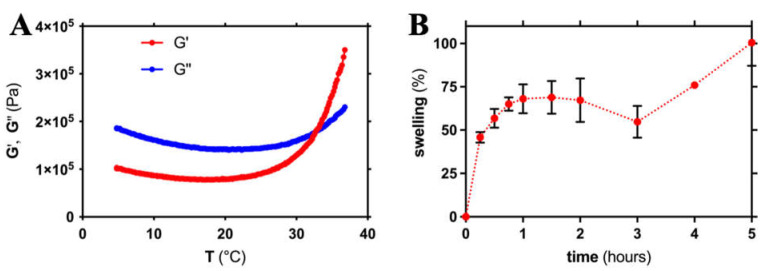
(**A**) Dependence of the elastic (G′) and viscous (G″) moduli on temperature for the mixture of chitosan and β-glycerophosphate. (**B**) Swelling behavior of hydrogel based on chitosan and β-glycerophosphate in PBS (pH = 7.4).

The gelation process was investigated by rheological tests. In detail, the loss tangent was recorded as a function of temperature (Figure 2A). The progressive increase in elastic modulus (G’) and decay of viscous modulus (G’’) suggested the transition from a viscous solution towards a hydrogel with time. Indeed, heat promotes the transfer of protons from positively charged chitosan chains (due to the positively charged amine groups) to β-glycerophosphate, progressively enhancing chitosan chain interaction and hydrogel formation [31]. The gelation time was calculated as the intersection of the two moduli [32] and was equal to 32.6 °C. This gelation temperature is optimal to devise injectable hydrogels to be used in regenerative medicine approaches. Indeed, this system can be easily mixed at a low temperature and injected in the recipient site to form a hydrogel in physiological conditions, at a temperature close to 37 °C. Similar gelation temperatures were previously reported for other systems based on chitosan and β-glycerophosphate [33,34,35]. On the other hand, other authors reported a different gelation temperature (from 21 to 79 °C) for systems based on chitosan and polyol phosphates (e.g., glucose-1-phosphate and glucose-6-phosphate) [32,36,37,38]. These differences can be attributed to the different concentrations of chitosan and polyol phosphates and also to the different behavior of the polyol phosphates [37].

The ability of the hydrogels to uptake solvent was investigated by swelling tests. The hydrogels showed a marked ability to uptake a large amount of solvent (Figure 2B). Specifically, after one hour, the hydrogels were able to increase their mass by 68.0 ± 8.3%, and after 5 h, their mass was doubled (+100.5 ± 13.4%).

Liposomes (0.96 mg/mL) containing cholesterol and α-phosphatidylcholine were prepared and characterized by dynamic light scattering (DLS) analyses. The liposomes had a medium hydrodynamic diameter close to 130 nm, associated with low dimensional dispersion (PDI lower than 0.2) (Table 1). Additionally, the liposomes displayed a negative surface charge. Similar dimensions and dimensional dispersions were reported by other authors for natural EVs [39,40,41]. This suggests that liposomes can be considered a simplified and synthetic model of native EVs.

In order to study the liposomes’ interactions with cells, fluorophore-labeled liposomes were also prepared. In this case, a green fluorescent derivative of lyso phosphatidylcholine—i.e., TopFluor^®^ Lyso PC, an analogue to α-phosphatidylcholine—was introduced during liposome synthesis to promote its integration within liposomes’ membrane [42]. By using this approach, the fluorescent derivative of the phospholipid was able to integrate within the liposome membrane, favored by both hydrophilic and hydrophobic interactions. The resulting liposomes showed a slight increase in size compared to non-labeled vesicles (Table 1). Specifically, fluorophore-labeled liposomes displayed dimensions of 179 ± 1 nm. The slight increase in dimensions indicates that the presence of the fluorescent lipid promoted limited aggregation and an increase in dimensional dispersion (PDI of 0.35 ± 0.02) of liposomes. 

### 2.2. In Vitro Characterization of the Hybrid System

The hybrid system was prepared by mixing liposomes and chitosan solution and subsequently promoting chitosan gelation by adding β-glycerophosphate. In order to investigate the interaction between liposomes and chitosan, a simplified model of the hybrid system was investigated. Highly concentrated—and viscous—chitosan solutions are not suitable for DLS analyses. In this case, a low chitosan concentration was exploited, and the resulting system—mentioned as chitosan-coated liposomes (0.98 mg/mL)—was analyzed by DLS. In the presence of chitosan, an increase in size was detected. Specifically, in the presence of chitosan, the medium hydrodynamic diameter was close to 240 nm (Table 1). This size increase was also associated with an opposite surface charge as this formulation displayed a positive surface charge. These phenomena suggest that the positively charged chitosan is able to associate to negatively charged liposomes, promoting new system formation with an increased size and a positive charge.

**Figure 3:**
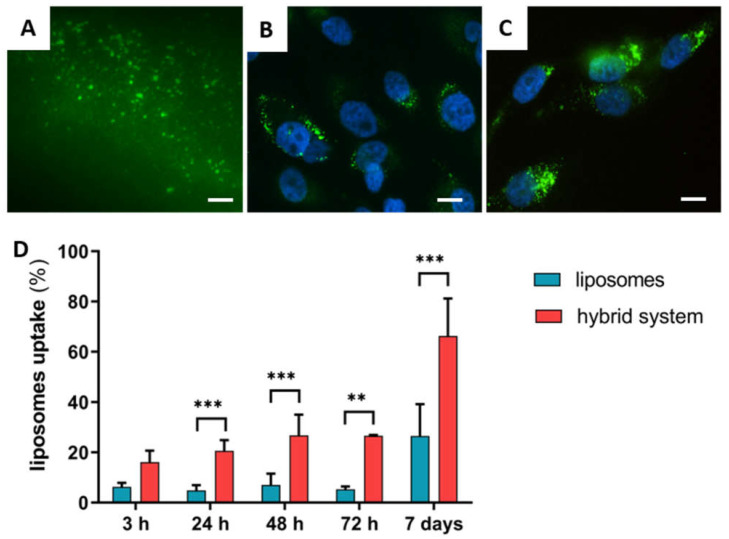
Fluorescence microscopy analyses of hybrid hydrogels based on fluorophore-labeled (TopFluor^®^ Lyso PC) liposomes after 24 h with cell media (**A**). Fluorescence microscopy analyses of MG63 cells after 24 h of incubation with liposomes (**B**) and with the hybrid system (**C**). The scale bar stands for 50 μm (in (**A**)) and 20 μm (in (**B**,**C**)). Lipid uptake from MG63 cells at different time points, i.e., 3 h, 24 h, 48 h, 72 h, and 7 days (**D**). Spectrofluorimetric analyses were performed to assess the internalization of fluorophore-labeled (TopFluor^®^ PC) liposomes. ** *p*-value < 0.01; *** *p*-value < 0.001.

In vitro tests with cells were then performed to assess the interaction between the biomaterial and cells and to verify that the chitosan coating is able to enhance cell interaction. Free fluorophore-labeled liposomes and the hybrid system resulting from the combination of aforementioned liposomes and the chitosan-based thermosensitive hydrogel were used to this end. After cell culturing for 24 h, the resulting samples were embedded in OCT, a high-viscosity medium to quickly embed fresh samples; frozen; and cut into 6 µm width slices using a cryostat. Samples were then analyzed with an inverted fluorescent microscope. The same procedure was performed on the hybrid thermosensitive hydrogel embedded with fluorophore-labeled liposomes and incubated with cell culture media—and without cells—for 24 h prior to the processing. By using the hybrid system even after the incubation in the media for 24 h, it was possible to detect the presence of liposomes within the hydrogel network mesh (Figure 3A). This result suggests that by exploiting this hybrid system, it is possible to provide a controlled release of liposomes. The controlled release was mainly promoted by the hydrogels swelling. Indeed, the thermosensitive hydrogels showed a marked ability to uptake a large amount of solvent (Figure 2B). This indicates that the polymer mesh of the hydrogel progressively tends to become loose and to promote a controlled release of liposomes. Figure 3B,C show how liposomes were efficiently internalized by cells within 24 h and how cells internalized a higher number of liposomes by using the hybrid system (Figure 3C).

The liposomes’ interaction with cells was then quantitatively investigated by spectrofluorimetric analyses: cells were incubated with fluorophore-labeled (TopFluor^®^ PC) liposomes; then, at different time points—from 3 h to 7 days—the uptake was determined (Figure 3D). Both free liposomes and the hybrid system were used. At all the time points investigated—3, 24, 48, and 72 h and 7 days—the percentage of liposomes internalized by cells was higher in the presence of the hybrid system compared to the use of free liposomes. In detail, in the hybrid system, a progressive increase in liposome uptake by cells was detected over time. More than 60% of liposomes (66.3 ± 15.0%) were internalized by cells after 7 days. On the other hand, when using free liposomes, the number of internalized liposomes was lower and constant up to 3 days (<10%) and then slightly increased at day 7 of incubation, with only around 30% of the liposomes internalized (26.5 ± 12.7%). 

**Figure 4:**
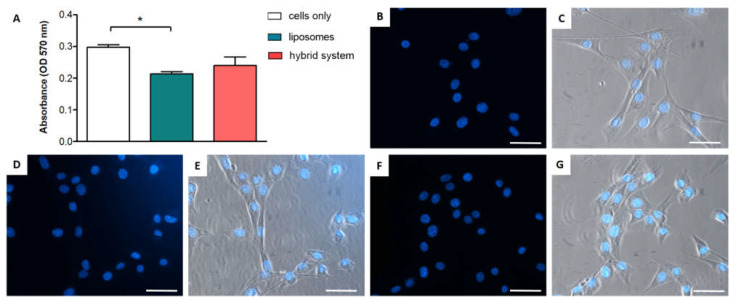
(**A**) Biocompatibility test by MTT assay after 24 h of incubation with liposomes and the hybrid system; cells without any treatment were used as a control. * *p*-value < 0.05. Fluorescence and merged (combined with bright field) microscopy analyses of fibroblasts (BALB/3T3 cells) after 24 h for control cells (**B**,**C**), cells incubated with liposomes (**D**,**E**), and cells incubated with the hybrid system (**F**,**G**). In all figures, the scale bar is 50 μm.

In vitro biocompatibility tests were then performed on free liposomes and the hybrid system (i.e., combination of liposomes and thermosensitive hydrogel). To this end, cell viability was verified by MTT assay after 24 h of incubation. While there was a slight reduction in cell viability in the presence of free liposomes, the hybrid system did not show any cytotoxicity; no statistical differences between the controls (cells only) and the cells treated with the hybrid system were detected (Figure 4A), suggesting the absence of toxicity in the formulation. This indicates that the hybrid system based on liposomes within a thermosensitive hydrogel can be considered suitable for cell culture.

A qualitative cell morphology analysis was then considered to verify the cell health and the absence of apoptotic cells. The cell nuclei morphology was analyzed by DAPI as fluorescent staining on an inverted fluorescent microscope. All nuclei display a defined round shape, without any debris and any apoptotic morphology, in either control cells (Figure 4B), in cells incubated with liposomes (Figure 4D), or with the hybrid system (Figure 4F). In all cases, no condensed chromatin aggregates were detected, confirming that no apoptotic cells were present [43].

Additionally, bright field images were simultaneously acquired (and superimposed to fluorescent images) in order to investigate the cell morphology. Control cells (Figure 4C) and cells incubated with liposomes (Figure 4E) or with the hybrid system (Figure 4G) displayed an elongated and healthy morphology, without any cellular debris. These results confirm the absence of toxicity of the present formulation. 

## 3. Discussion

In the present study, we have proposed a method to fabricate a hybrid system based on liposomes and a thermosensitive hydrogel for regenerative medicine applications. A hybrid system is able to overcome some drawbacks commonly shown with the use of free liposomes (e.g., rapid clearance and limited therapeutic potential) [5,6,7]. Among all the injectable and biocompatible systems suitable for regenerative medicine applications, chitosan and β-glycerophosphate were selected for their peculiar properties. Chitosan is a biocompatible, biodegradable, and non-immunogenic polymer derived from the seafood waste industry [23,29]. This polymer is very versatile and can be used to fabricate different structures, including fibers, hydrogels, scaffolds, and micro- and nanoparticles [44,45,46,47]. These systems based on chitosan are widely used for biomedical applications, especially for the delivery of therapeutic agents and for regenerative applications [45,48,49,50,51]. On the other hand, β-glycerophosphate is a biocompatible and naturally occurring molecule that is commonly present in our body. More specifically, β-glycerophosphate serves as a source for the phosphate in hydroxyapatite synthesis and is able to tune the differentiation of target cells, including the differentiation of stem cells toward an osteogenic phenotype [31,52].

The hydrogel investigated in this study showed a gelation temperature equal to 32.6 °C, indicating the possibility to be used as an injectable delivery system. By combining the thermosensitive hydrogel with liposomes, we obtained a hybrid controlled-release system. This system can be applied to achieve sustained release of liposomes or EVs, which normally show a poor half-life when administrated alone [14,17,18]. Although liposomes have a synthetic origin and EVs are naturally produced by cells, both are lipid bilayer nanocarriers able to deliver bioactive molecules, so liposomes may be considered a simplified EV model [3,13].

Despite a slight reduction in cell viability in the presence of free liposomes, the hybrid system did not show any cytotoxicity. A similar reduction in the cell viability of a human carcinoma cell line (A549 cells) in the presence of free liposomes was previously reported by other authors for cationic liposomes and was attributed to their peculiar surface charge [53]. A comparable reduction in cell viability was also reported by Soe and colleagues for the same cell line incubated with free liposomes [54]. On the other hand, the present hybrid system based on liposomes and the thermosensitive hydrogel did not show any cytotoxicity towards a cell model. In this case, liposomes’ cell internalization resulted to be strictly affected by their features: chitosan was able to efficiently interact with liposomes, promoting a change—from negative to positive—in the surface charge. This change in physicochemical properties was attributed to the efficient ability of positively charged chitosan to interact with negatively charged phospholipids of liposomes, concealing their native surface charge and bestowing a positive surface charge to chitosan-coated liposomes. In vitro tests suggested that the presence of chitosan is able to enhance the interaction with cells and to promote a higher cellular uptake. In fact, the liposomes were progressively internalized by the cells in a controlled manner at higher concentrations compared to free liposomes. This ability can be attributed to electrostatic interactions between the positively charged chitosan and the negatively charged liposomes’ membrane [55,56]. Thus, liposomes coated with chitosan were able to efficiently interact with cells. Furthermore, chitosan is able to efficiently interact with the membrane receptor CD44, which is overexpressed in many cell types [57,58]. Consequently, liposomes in the presence of chitosan were efficiently internalized by cells.

Overall, this study demonstrated the possibility of combining a thermosensitive hydrogel and liposomes. Considering all the benefits offered by the resulting system, we propose the use of these hydrogels as a versatile platform that can be translated into the controlled release of EVs, and they can be used as innovative translational biomaterials in the field of regenerative medicine.

## 4. Materials and Methods

### 4.1. Materials

Low-molecular-weight chitosan (CH, 50,000–190,000 Da), β-glycerophosphate disodium salt hydrate (β-GP), cholesterol, acetic acid, α-phosphatidylcholine (α-PC), TopFluor^®^ lysophosphatidylcholine, thiazolyl blue tetrazolium bromide (MTT), paraformaldehyde (PFA), phosphate-buffered saline (1X) w/o Ca and Mg (PBS) (pH = 7.4), sodium tripolyphosphate (TPP), dimethyl sulfoxide (DMSO), and absolute ethanol were purchased from Merck KGaA (Darmstadt, Germany). 4’,6-diamidino-2-phenylindole, dihydrochloride (DAPI) reagent, fetal bovine serum (FBS), penicillin/streptomycin mixture (pen/strep), and Dulbecco’s Modified Eagle Medium (DMEM) and Dulbecco’s Modified Eagle Medium nutrient mixture F12 (DMEM/F12) were from ThermoFisher Scientific (Waltham, MA, USA). Optimal cutting temperature (OCT) compound was from Histo-Line Laboratories (Pantigliate, Italy).

### 4.2. Hydrogels Preparation

Chitosan and β-glycerophosphate solutions were separately prepared. The concentration of chitosan was equal to 25 mg/mL, whereas the concentration of β-glycerophosphate was equal to 645 mg/mL. Chitosan was solubilized in deionized water containing 1% *v*/*v* acetic acid, whereas β-glycerophosphate was solubilized in deionized water. Both solutions were stored overnight at 4 °C before mixing. Then, β-glycerophosphate solution was added dropwise to the chitosan solution in an ice bath (to maintain a stable temperature) under magnetic stirring. The resulting mixture was incubated overnight at 37 °C to promote the hydrogel formation.

### 4.3. Hydrogel Characterization

Rheological measurements were performed using a Bohlin C-VOR 120 rotational rheometer equipped with a thermostatic unit (KTB 30). After samples’ preparation (according to Section 4.2), the resulting mixture at 4 °C was transferred onto the rheometer plate. The experimental settings used to evaluate gelation kinetics are the following: stainless steel plates with 4° cone/plate geometry, diameter = 40 mm, and gap = 0.150 mm. Temperature sweep experiments were performed in strain-controlled conditions, with deformation (γ) of 0.01 and frequency (ν) of 1 Hz kept constant throughout the experiment. Upon addition of β-glycerophosphate, samples were mixed under stirring for about 10 s to make them uniform and then poured on the plate. The values of storage G’ (elastic response) and loss G’’ (viscous response) moduli were recorded as a function of time. Temperature sweep experiments were performed in the range of 5–37 °C.

Silicone oil (viscosity 50 cSt, purchased from Sigma, Saint Louis, MO, USA) was used to seal the interface between the two plates in order to improve thermal control and limit solvent evaporation.

### 4.4. Swelling Tests on Hydrogels

The resulting hydrogels were transferred in PBS at T = 37 °C. At selected time points, the hydrogels were removed from the wells and weighed in order to evaluate hydrogels’ swelling. Data are reported as % of mass gained with respect to the initial weight of three samples (± standard deviation, SD).

### 4.5. Liposomes Preparation

Cholesterol and α-PC were solubilized by using ethanol as a solvent. The concentration of cholesterol was equal to 20 mg/mL, whereas that of α-PC was equal to 40 mg/mL. The organic phase consisted of 25 µL of cholesterol solution, 12.5 µL of α-PC solution, and 62.5 µL of ethanol (total volume 0.1 mL), while the aqueous phase consisted of 1 mL of deionized water. For liposomes’ preparation, the organic phase was injected (1-milliliter syringe, 25 G) in the aqueous phase at room temperature under magnetic stirring (700 rpm). The resulting mixture was maintained under stirring (250 rpm) for 5 min. After 30 min, liposomes were collected.

Fluorophore-labeled liposomes were prepared by exploiting TopFluor^®^ Lyso PC, an analogue to α-phosphatidylcholine. To this end, this fluorophore-labeled phospholipid (final concentration 57 μg/mL) was added during liposome synthesis. A calibration curve for quantification in fluorescence was prepared in the range of 0.05–1 μg/mL (R^2^ > 0.99).

### 4.6. Hybrid System Preparation

The hybrid system was prepared by mixing liposomes (prepared according to Section 4.5) and chitosan solution (1% *v*/*v* acetic acid as solvent) at 4 °C. The volume ratio between liposomes and chitosan solution was equal to 1:4.56. β-glycerophosphate was then added to the mixture according to Section 4.2. The same abovementioned final concentrations of chitosan and of β-glycerophosphate were used.

### 4.7. Dynamic Light Scattering

Chitosan-coated liposomes were prepared in order to investigate the interaction between liposomes and chitosan. Indeed, highly viscous solutions and macroscopic hydrogels are not suitable for DLS analyses. To this end, the aqueous phase of liposomes was substituted by TPP solution (1.5 mg/mL). The resulting liposomes (1.1 mL) were then added dropwise to a chitosan solution (1 mg/mL in 0.1% *v*/*v* acetic acid solution, 1 mL) under magnetic stirring (250 rpm) at room temperature for 30 min.

Liposomes and chitosan-coated liposomes were characterized by means of dynamic light scattering (DLS) with a Zetasizer Nano ZS with 173° detection optics (Malvern Instruments, USA). The dimensions (hydrodynamic diameter), polydispersity index (PDI), and surface charge (ξ-potential) of the formulations were investigated. Formulations were analyzed in triplicate.

### 4.8. In Vitro Biological Tests

BALB/3T3 cells (ATCC CCL-163) were used for cytotoxicity tests. BALB/3T3 cells were grown using DMEM with 10% *v*/*v* FBS and 1% *v*/*v* penicillin/streptomycin. MG63 cells (human osteosarcoma cells, CRL-1427, ATCC^®^, Washington, DC, USA) were used to evaluate the cell interaction. MG63 cells were grown using DMEM/F12 medium with 10% *v*/*v* FBS and 1% *v*/*v* penicillin/streptomycin.

#### 4.8.1. *In Vitro* Cytotoxicity Tests

BALB/3T3 cells were seeded in a 96-well plate with 2500 cells/cm^2^ density. After 24 h, the hybrid formulation was diluted in cell culture medium (dilution factor equal to 20) and added to the wells. Cells without any additional treatment were used as a control. After 24 h, the MTT test was used for the evaluation of cells’ biocompatibility. Briefly, a small volume of MTT solution (5 mg/mL) in PBS 1X was added in 1/10 ratio to the wells. Cells were incubated for 2 h at 37 °C and then the cell culture medium was removed. Finally, DMSO was added to each well (0.2 mL). After 15 min, supernatants were analyzed using a UV-visible spectrophotometer (Multiskan FC, Thermo Scientific, Waltham, MA, USA) at 570 nm absorbance. Additionally, cell nuclear morphology was analyzed by DAPI staining after cell fixation in 4% buffered paraformaldehyde (PFA) and microscope visualization with an inverted fluorescent microscope (Eclipse TS 100, Nikon, Japan).

#### 4.8.2. Cell Interaction

For indirect release study in vitro, MG63 cells (human osteosarcoma cells, ATCC^®^ CRL-1427) were seeded at 2500 cell/cm^2^ density. After 24 h, hybrids were added to cells after dilution in cell medium (dilution factor equal to 10). After 1 day, cell media were collected and centrifuged (13,800× *g*, 5 min). The resulting pellets were included in OCT and frozen in liquid nitrogen. Serial sections of 6 μm width were obtained using a semi-automatic cryostat (MC500, Histo-line Laboratories, Pantigliate, Italy) and visualized with an inverted fluorescent microscope. Cells were fixed in 4% PFA, and the nuclei were stained with DAPI. Finally, cells were visualized with an inverted fluorescent microscope. For fluorescent lipid quantification assays, liposomes and hybrids were loaded with 56.8 and 11.4 µg/mL of fluorescent lipid, respectively. Cells were seeded in a 96-well plate at 1500 cells/well density. After 24 h, formulations were 10-fold diluted in cell media and incubated with cells. At fixed time points (3 h, 24 h, 48 h, 72 h, 7 days), fluorescent lipid uptake was evaluated by spectrofluorimetric analyses (Fluoroskan FL, Thermo Scientific, USA).

### 4.9. Statistical Analysis

Statistical analysis and graph elaboration were performed using GraphPad Prism 9.0.0 (GraphPad Software, San Diego, CA, USA). Two-way ANOVA (analysis of variance) was performed followed by Dunnett’s multiple comparisons test to evaluate differences among different groups and the control. An unpaired Student’s *t*-test was performed to evaluate differences between two groups. Differences were considered significant for *p*-values less than 0.05 (* *p*-value < 0.05; ** *p*-value < 0.01; *** *p*-value < 0.001).

## 5. Conclusions

In the present work, two different systems were combined in order to devise an injectable nanocomposite system for the controlled release of liposomes for regenerative medicine applications. The resulting system was able to provide controlled release of liposomes, preventing the disadvantages related to the use of free liposomes. The controlled release was promoted by the hydrogel swelling. Indeed, the thermosensitive hydrogels showed a marked ability to uptake a large amount of solvent. This indicates that the polymer mesh of the hydrogel progressively tends to become loose and promote controlled release of liposomes.

Additionally, by using fluorescent labeling of liposomes, it was possible to investigate the interaction of free liposomes and chitosan-coated liposomes with cells. The presence of a chitosan coating was able to change the physicochemical properties of liposomes—promoting the change of surface charge from negative to positive—and to enhance their interaction with cells. This latter aspect is of pivotal importance and highlights that chitosan can be used to improve the interaction with cells for a large number of possible applications—not only limited to regenerative medicine. We propose the use of these hydrogels as a versatile platform that can be translated into the controlled release of EVs, and they can be used for novel and advanced applications in regenerative medicine. This system can also be easily adapted for the controlled release of different nucleic acids, bioactive molecules (e.g., natural compounds for nutraceutical applications), and drugs.

## Figures and Tables

**Table 1:** Dimensions, polydispersity index (PDI), and surface charge (ξ-potential) values of liposomes and chitosan-coated liposomes (all of them reported as medium value ± SD of three measurements).

Sample	Dimensions (nm)	Polydispersity Index (PDI)	Surface Charge (ξ-Potential)
Liposomes	133 ± 25	0.15 ± 0.02	−21 ± 6
Chitosan-coated liposomes	236 ± 43	0.33 ± 0.05	33 ± 3

## Abbreviations

4’,6-diamidino-2-phenylindole, dihydrochloride (DAPI); α-phosphatidylcholine (α-PC); β-glycerophosphate disodium salt hydrate (β-GP); dimethyl sulfoxide (DMSO); dynamic light scattering (DLS); Dulbecco’s Modified Eagle Medium (DMEM); Dulbecco’s Modified Eagle Medium nutrient mixture F12 (DMEM/F12); extracellular matrix (ECM); extracellular vesicles (EVs); fetal bovine serum (FBS); low-molecular-weight chitosan (CH, 50,000–190,000 Da); optimal cutting temperature (OCT) compound; paraformaldehyde (PFA); phosphate-buffered saline (1X) w/o Ca and Mg (PBS); polydispersity index (PDI); sodium tripolyphosphate (TPP); thiazolyl blue tetrazolium bromide (MTT).
